# Effect of Serotype and Strain Diversity on Dengue Virus Replication in Australian Mosquito Vectors

**DOI:** 10.3390/pathogens9080668

**Published:** 2020-08-18

**Authors:** O’mezie Ekwudu, Louise Marquart, Lachlan Webb, Kym S. Lowry, Gregor J. Devine, Leon E. Hugo, Francesca D. Frentiu

**Affiliations:** 1 Institute of Health and Biomedical Innovation, School of Biomedical Sciences, Queensland University of Technology, Brisbane 4000, Australia; o.ekwudu@qut.edu.au (O.E.); k.lowry@uq.edu.au (K.S.L.); 2 Mosquito Control Laboratory, QIMR Berghofer Medical Research Institute, Brisbane 4006, Australia; Greg.Devine@qimrberghofer.edu.au (G.J.D.); Leon.Hugo@qimrberghofer.edu.au (L.E.H.); 3 Department of Microbiology, Chukwuemeka Odumegwu Ojukwu University, Uli 431124, Nigeria; 4 Statistics Unit, QIMR Berghofer Medical Research Institute, Brisbane 4006, Australia; Louise.Marquart@qimrberghofer.edu.au (L.M.); Lachlan.Webb@qimrberghofer.edu.au (L.W.); 5 Clinical Malaria, QIMR Berghofer Medical Research Institute, Brisbane 4006, Australia

**Keywords:** dengue viruses (DENV), *Aedes aegypti*, *Aedes albopictus*, arbovirus, flavivirus, vector competence, virus kinetics, genetic diversity

## Abstract

Dengue virus (DENV) is the most important mosquito-borne viral pathogen of humans, comprising four serotypes (DENV-1 to -4) with a myriad of genotypes and strains. The kinetics of DENV replication within the mosquito following ingestion of a blood meal influence the pathogen’s ability to reach the salivary glands and thus the transmission potential. The influence of DENV serotype and strain diversity on virus kinetics in the two main vector species, *Aedes aegypti* and *Ae. albopictus*, has been poorly characterized. We tested whether DENV replication kinetics vary systematically among serotypes and strains, using Australian strains of the two vectors. Mosquitoes were blood fed with two strains per serotype, and sampled at 3, 6, 10 and 14-days post-exposure. Virus infection in mosquito bodies, and dissemination of virus to legs and wings, was detected using qRT-PCR. For both vectors, we found significant differences among serotypes in proportions of mosquitoes infected, with higher numbers for DENV-1 and -2 versus other serotypes. Consistent with this, we observed that DENV-1 and -2 generally replicated to higher RNA levels than other serotypes, particularly at earlier time points. There were no significant differences in either speed of infection or dissemination between the mosquito species. Our results suggest that DENV diversity may have important epidemiological consequences by influencing virus kinetics in mosquito vectors.

## 1. Introduction

Dengue virus (DENV) is one of the most important arthropod-borne viruses affecting humans [1]. It is a positive-sense RNA virus and member of the Flavivirus genus, comprising four antigenically distinct serotypes with multiple genotypes and strains within these. Dengue outbreaks in Australia predate the 20th century [2], but the past two decades have seen an increasing number of outbreaks related to increased international air travel and the consequent importation of viruses with travellers returning to Australia from endemic countries [3,4,5]. Local virus transmission occurs in central and North Queensland, where there is an established presence of *Aedes aegypti*, the primary vector of DENV in Australia [5,6]. The increasing number and frequency of DENV importations heightens the chances of local human populations being exposed to multiple serotypes of DENV and the risk of severe disease [7]. *Aedes albopictus* is a highly invasive species with an expanding worldwide range [8], and is also a competent vector of DENV and other arboviruses [9,10]. It thrives in both temperate and tropical climates [11]. In the Australian context, modelling has shown that all of mainland Australia’s coastal regions would provide suitable habitat for *Ae. albopictus* should it invade [12]. If this were accompanied by an expanding geographic distribution of DENV, seasonal epidemics in urban centres like Brisbane or Perth would result in major public health burdens [13]. Populations of *Ae. albopictus* have already become established in the Torres Strait between mainland Australia and Papua New Guinea, most likely due to human maritime traffic from Indonesia [12,14]. The species has been intercepted at Australia’s mainland air and seaports [15] and was implicated in a 2016 dengue outbreak in the Torres Strait [16].

The first dengue vaccine Dengvaxia^®^ is licensed in some countries but, due to adverse safety events [17], the vaccine is recommended only for persons having a prior history of dengue infection, who are between 9 and 45/60 years and live in a dengue endemic region [18]. Without a universally available vaccine, efforts to prevent dengue continue to rely on mosquito control strategies, which can include the use of physical barriers, chemical insecticides and biocontrol [19,20]. Many of these efforts have been ineffective at times, due, in part, to an incomplete understanding of the factors that regulate DENV transmission by mosquitoes.

Mosquito populations from different geographic locations vary in their susceptibility to infection with, as well as the ability to transmit, different strains of DENV [21,22]. Variability among DENV serotypes and lineages within genotypes has been associated with differences in the extrinsic incubation period (EIP) [23,24,25], the time it takes an arbovirus to disseminate in the mosquito, from the ingestion of an infected blood meal to when the virus can be detected in saliva. Previous studies have reported variation in DENV EIPs of between 2 and 15 days [26]. For example, differences in the dissemination of different strains of DENV-2 have been observed [27] and a Southeast Asian genotype of DENV-2 has been shown to have shorter EIP than an American strain which it displaced [28]. However, most vector competence studies have focused on the susceptibility of mosquito populations to infection with individual strains or small collections of strains that represent only a subset of the genetic diversity of the dengue viruses [29,30]. This reduces the accuracy of comparison of virus replication kinetics between serotypes as virus replication is highly dependent on experimental factors. Furthermore, investigations are typically restricted to assessments of virus kinetics within a single mosquito species [31]. Here, we systematically tested whether variation in DENV serotypes and strains results in differences in infection and dissemination kinetics by simultaneously testing representative strains from all four dengue serotypes in both *Ae. aegypti* and *Ae. albopictus*.

## 2. Results

We quantified the replication dynamics of eight genetically diverse strains of DENV representing all four serotypes (two strains/serotype), that had been isolated from outbreaks in South East Asia and the Pacific over a 25-year period (Table 1). We orally exposed mosquitoes from Australian populations of *Ae. aegypti* and *Ae. albopictus* to 10^7^ pfu/mL of DENV in sheep blood. The overall proportions of *Ae. aegypti* (63%) and *Ae. albopictus* (57%) that engorged on the DENV infected blood meals were similar.

Mosquitoes were sampled at 3, 6, 10 and 14 d post exposure (dpe) and tested for DENV infection in bodies and dissemination to legs and wings (hereafter referred to as legs/wings) using quantitative Reverse Transcriptase—Polymerase Chain Reaction (qRT-PCR). Both species of mosquito were susceptible to infection with all DENV strains tested.

The proportion of *Ae. aegypti* bodies with a detectable DENV infection increased over time for each strain (Table 2). Infection rate patterns for strains within serotypes were broadly similar and differed between DENV serotypes. DENV serotypes 1 and 2 strains infected the highest proportion of mosquitoes (range 35–70% at 14 dpe) whereas strains from serotypes 3 and 4 infected a maximum of 30% of mosquitoes by 14 dpe. DENV-2 strain NC-483 infected the highest proportion of mosquitoes of any strain (70%). Dissemination of DENV to *Ae. aegypti* mosquito leg and wing tissue was first detected at 6 dpe for all serotypes (Table 2). The proportion of mosquitoes with virus in leg and wing tissues gradually increased over time, reaching a maximum of 20–40% of mosquitoes for DENV-1 and DENV-2 strains, and 10–20% for DENV-3 and DENV-4 strains. Given the similarities between strains from each serotype, the data were pooled for each serotype and the effect of serotype on the proportion of mosquitoes infected at different time points was tested (Supplemental Table S1). Serotypes differed significantly in their ability to lead to a detectable infection in mosquitoes at 6 and 10 dpe (*p* < 0.05, Fisher’s exact test). When leg and wing infection rates were pooled for different strains within each dengue serotype, serotype significantly affected the proportion of mosquitoes with a leg and wing infection at 10 dpe but not at the other time points (Supplemental Table S1).

**Figure 1:**
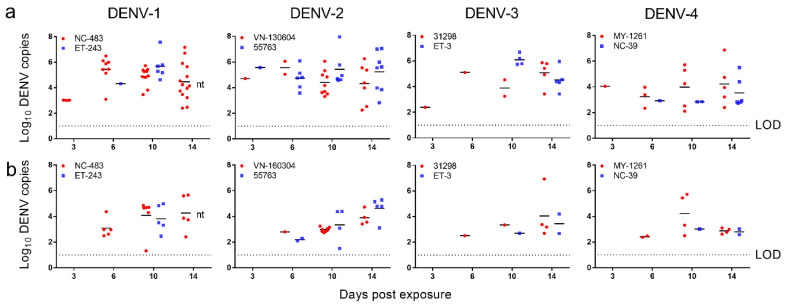
Growth kinetics of dengue virus strains from all four serotypes within *Ae. aegypti* mosquitoes. Virus loads in (**a**) bodies and (**b**) legs and wings. Points show the number of virus copies in individual samples. Lines indicate means. nt, not tested (mosquitoes infected with DENV-1 ET-243 did not survive to 14 dpe). LOD, limit of detection.

The levels of DENV RNA accumulation in *Ae. aegypti* bodies and legs/wings harvested after the four incubation periods were quantified by qRT-PCR. Virus loads in mosquito bodies and legs/wings samples, defined here as the log_10_ number of DENV copies per sample, were determined for individual *Ae. aegypti* mosquitoes according to virus strain and time point (Figure 1). The highest mean (±SD) virus load in bodies was from DENV-3 (ET-3), reaching 6.10 ± 0.44 log_10_ DENV copies at 10 dpe (Figure 1a). The number of virus copies detected from *Ae. aegypti* leg and wing tissue generally increased or remained stable from 6 to 14 dpe and reached between 2.3 to 7 log_10_ genome copies across all strains at 14 dpe (Figure 1b). The highest mean (±SD) virus load in legs and wings was from DENV-2 55763, which reached 4.62 ± 0.87 log_10_ RNA copies/mosquito at 14 dpe.

Similar to *Ae. aegypti*, the proportion of *Ae. albopictus* bodies with a detectable dengue infection increased over time and infection patterns differed more greatly between dengue serotypes than for strains within each serotype (Table 2). Higher infection rates were again observed from DENV-1 and DENV-2 strains (20–55% mosquitoes infected) compared to DENV-3 and DENV-4 strains (10–45% mosquitoes infected). The data were pooled for each serotype and significant differences in the proportion of mosquito bodies with a detectable dengue infection were observed between serotypes at 6, 10 and 14 dpe (Supplemental Table S2). Disseminated infections were first detected from *Ae. albopictus* leg and wing tissue between 6–10 d for DENV-1 and DENV-2 strains and between 6–14 d for DENV-3 and DENV-4 strains (Table 2). The proportion of mosquitoes with detectable infection in *Ae. aegypti* leg and wing tissue generally increased over time to reach a maximum of between 10 and 30% of mosquitoes for DENV-1 and DENV-2 strains and between 5% and 20% for DENV-3 and DENV-4 (Table 2). The proportion of mosquitoes with detectable infection in leg and wing tissue differed significantly between dengue serotypes at 6 and 10 dpe but not at the other time points (Supplemental Table S2).

**Figure 2:**
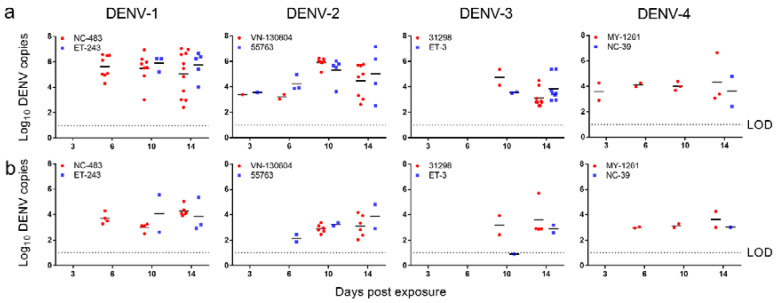
Growth kinetics of dengue virus strains from all four serotypes within *Ae. albopictus* mosquitoes. Virus loads in (**a**). bodies and (**b**). legs and wings. Points show the number of virus copies in individual samples. Lines indicate means. LOD, limit of detection.

The mean number of dengue genome copies in *Ae. albopictus* bodies generally increased from 0–10 dpe before stabilizing or decreasing to 14 dpe (Figure 2). The highest mean (±SD) virus loads were observed from DENV-2 VN130604 and DENV-1 ET243 (5.91 ± 0.36 log_10_ and 5.90 ± 0.60 log_10_ RNA copies/mosquito, respectively, at 10 dpe). The mean number of dengue genome copies in leg and wing tissue generally remained stable between 6 and 14 d, however there was an increasing trend for DENV-2. The highest mean (±SD) DENV titre in *Ae. albopictus* legs and wings was observed from DENV-1 (NC-483) at 14 dpe (4.27 ± 0.41 log_10_ RNA copies/mosquito).

## 3. Effects of Mosquito Species, Virus Strain and Incubation Time on DENV Loads

Given the strong similarities in the infection kinetics of the DENV strains in both *Ae. aegypti* (Figure 1) and *Ae. albopictus* (Figure 2), we tested the significance of the main effects of mosquito species, virus strain and incubation time using a general linear model (GLM). The main effects of incubation time and virus strain significantly affected genome levels for body and legs/wings samples (Table 3). However, the main effect of mosquito species (*Ae. aegypti* or *Ae. albopictus*) was not significant for either tissue type.

**Figure 3:**
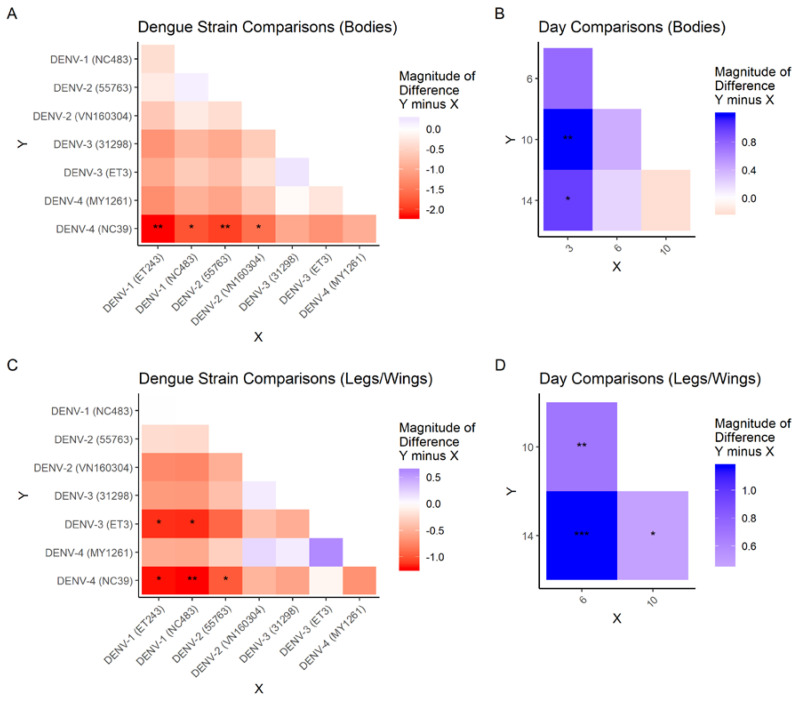
Magnitude of differences from Tukey’s pairwise difference test for each pair of dengue strain and pair of days post DENV exposure (Days). Direction of difference in reference to Y axis (vertical) minus X axis (horizontal). (**A**) Pairwise comparisons among Dengue strains in Bodies, (**B**) Pairwise comparisons among Days in Bodies, (**C**) Pairwise comparisons among Dengue strains in Legs/Wings, (**D**) Pairwise comparisons among Days in Legs/Wings. *, *p* < 0.05. **, *p* < 0.01. ***, *p* < 0.001.

As the overall effect of mosquito species was not significant, data for both species were pooled and the within-treatment effects of virus strain and incubation time were compared for bodies and legs/wings by pairwise testing using Tukey’s method. DENV-1 and DENV-2 reached significantly higher virus genome copy numbers in mosquito bodies than the DENV-4 strain NC-39s (Tukey’s multiple pairwise comparisons; Figure 3). DENV-1 strains and DENV-2 (55763) reached higher genome copy numbers in mosquito legs and wings than mosquitoes infected with DENV-4 NC39, while all other strains reached intermediate titres in legs/wings. Similarly, over all strains and mosquito species, DENV virus titres in mosquito bodies increased significantly from 3 dpe to 10 dpe (Tukey’s multiple pairwise comparisons; Figure 3) before stabilising. In legs and wings, viral RNA was not detected in any mosquito at 3 dpe, however there were significant increases in virus genome copy numbers over successive time points from 6 to 14 dpe.

## 4. Discussion

We have identified substantial differences in the kinetics of virus infection between dengue serotypes and strains within the two major mosquito vector species, *Ae. aegypti* and *Ae. albopictus*. Virus infection kinetics were generally more similar between strains within a serotype than strains from other serotypes. We observed that DENV-1 and -2 infected the largest proportion of mosquitoes and generally produced the highest titres within mosquito tissue, consistent with previous observations from studies utilizing independent virus strains [32] and genetically different mosquito populations [33]. These findings support a hypothesis that the higher infectivity and increased virulence of DENV-1 and DENV-2 in *Aedes* mosquito vectors have contributed to the global dominance and higher disease burden caused by DENV-1 and -2 [34]. However, some within serotype variation was observed. For example, while DENV-1 NC483 out-performed most of the other DENV strains in both infection and dissemination, DENV-1 ET243 disseminated at a relatively slower rate, especially in *Ae. albopictus.* Furthermore, across all virus serotypes and strains, variation in virus load between mosquito species was non-significant. This challenges the reputation of *Ae. albopictus* as a secondary vector of dengue behind *Ae. aegypti.* Data on transmission rates are required to confirm these findings.

Most previous studies of virus replication dynamics and EIP for DENV have infected a single species of mosquito with individual or small collections of virus strains, making it difficult to compare virus replication kinetics between DENV serotypes [23,35,36]. As such, there is limited data available on the influence of DENV serotype or strain on the kinetics of DENV infection in mosquitoes [26]. Here we simultaneously evaluated multiple strains of the four serotypes of DENV. We show that both serotype and strain differences help determine the speed of dissemination of dengue virus within mosquitoes. In general, DENV-2 VN130604 achieved peak infection rate in both mosquito species at day 10 dpe while DENV-3 ET3 achieved peak infection and dissemination rates at 14 days post infection. This may be due to differences in virus replication kinetics that reflect intrinsic differences between virus serotypes.

Differences in the kinetics of infection in mosquitoes potentially affect mosquito vector competence and progression of dengue outbreaks [31]. Although it is well established that other factors, including temperature [37,38,39] and mosquito genes [40] influence virus replication dynamics, the impact of variation in serotypes and strains is often ignored in vector competence models [41]. Virus strain diversity contributes to heterogeneity in DENV transmission dynamics and while it may be impossible to evaluate individual strain attributes, an attempt to capture the evolutionary and epidemiological potential of different strains should be made in future dengue control strategies and models.

*Aedes albopictus* is considered to play a secondary role in transmission of DENV where *Ae. aegypti* and *Ae. albopictus* co-exist. Previous studies found that dissemination of dengue virus took longer in *Ae. albopictus* than in *Ae. aegypti* [33,42], however comparisons were not significant in our analyses (data not shown). Dissemination rates at 14 days post exposure varied from 10 to 50% in *Ae. aegypti* and from 5 to 45% in *Ae. albopictus,* which indicated that both species may become competent for transmission at a similar rate. Furthermore, we showed that the dengue strains reached equivalent titres in both vector species. *Ae. albopictus* has been implicated in DENV transmission in the Torres Strait [16] and should it become established on the Australian mainland, it would present a serious risk of DENV transmission in any urban centres that receive large numbers of viraemic visitors or returning residents from dengue endemic countries [12]. Key to gauging that risk is an understanding of the competence of *Ae. albopictus* for commonly circulating strains of DENV. Our findings, together with greater potential for range expansion of *Ae. albopictus* in Australia necessitate that *Ae. albopictus* be regarded as posing at least an equivalent level of risk of dengue transmission to *Ae. aegypti* in Australia.

A limitation of our study is that we did not investigate the appearance of detectable virus in the saliva of mosquitoes. However, previous evidence indicates that dissemination of DENV to legs and wings of mosquitoes is generally a reliable indicator of whether mosquitoes are capable of transmitting DENV [23,43,44], at least for *Ae. aegypti*. Another feature of this study which may affect how the outcome is compared to other assessments of mosquito vector competence is the use of qRT-PCR to quantify virus titre in mosquito tissues rather than a live virus assay such as a plaque assay to detect infectious virus. However, the detection of virus genomes allowed us to effectively determine the kinetics of infection such as the time at first detection of virus in different tissues and to measure relative differences in virus titres between the serotypes. We only tested a single infectious dose of 10^7^ PFU/mL. This dose is within the range of titres observed from human viraemia; however, a range of infectious doses may have presented a different set of parameter estimates given that infectious dose is a powerful determinant of DENV infection probability and virus kinetics in *Ae. aegypti* [45,46,47].

This study has established that there is significant variation in the interval between the ingestion of a blood meal and the appearance of detectable virus in the legs and wings of Australian *Ae. aegypti* and *Ae. albopictus* among DENV isolates representing the diversity of serotypes circulating in Southeast Asia and the Pacific. The heterogeneity in DENV transmission kinetics highlighted here, even within the same serotype, could potentially affect the timing and magnitude of dengue outbreaks. Should *Ae. albopictus* become established on mainland Australia, it is possible this species will be able to transmit multiple strains of DENV to a similar extent to *Ae. aegypti*. This work represents the first parallel evaluation of multiple regionally relevant strains of DENV in regionally relevant populations of *Ae. aegypti* and *Ae. albopictus* mosquitoes and raises important questions about the epidemiological implications of strain and serotype diversity in DENV. It also underscores the potential contribution of *Ae. albopictus* to DENV transmission in mainland Australia.

## 5. Materials and Methods

### 5.1. Viruses

Four strains of DENV were obtained from the WHO Collaborating Centre for Arbovirus Reference and Research at Queensland University of Technology (QUT) Australia, with additional strains being generous gifts from Prof Paul Young (The University of Queensland) and Dr Myrielle Dupont-Rouzeyrol (Institut Pasteur New Caledonia). All viruses had been passaged 3–7 times after isolation from patients. The viruses were propagated at 27 °C in mosquito C6/36 cells following infection at a multiplicity of infection of 0.1. Supernatants containing infectious virus were harvested at times of peak virus production; 5 days post infection (p.i.) for DENV-1 (NC-483), DENV-2 (55763), DENV-4 (NC-39) and DENV-4 (MY1261), at 6 days p.i. for DENV-2 (VN-130604) and DENV-3 (ET-3), and 7 days p.i. for DENV-1 (ET-243) and DENV-3 (31298). Virus stocks were then concentrated by ultrafiltration in 100 kDa Amicon filters (Merck Millipore, Burlington, MA, USA) according to the manufacturer’s instructions and aliquoted into sterile 2 mL tubes before freezing at −80 °C.

### 5.2. Mosquitoes

*Ae. aegypti* and *Ae. albopictus* mosquitoes used in infection experiments were sourced from established colonies at QIMR Berghofer Medical Research Institute (QIMR Berghofer). The *Ae. albopictus* colony originated from eggs collected on Hammond Island, Torres Strait in June 2014. The *Ae. aegypti* colony was established from *Wolbachia*-free eggs collected from Cairns, Queensland in 2015. Both mosquito colonies were established and maintained in the QIMR Berghofer insectary at 27 (±1) °C and 75 (±5)% relative humidity (RH), with a photoperiod of 12 h:12 h light:dark (L:D) cycles.

### 5.3. Immunofocus Assay

Titres of infectious virus were quantified by performing an immuno-focus assay based on the immuno-detection of infectious foci developing in African green monkey kidney epithelial cell (Vero: ATCC) monolayers. Once confluent, cells were inoculated with ten-fold serial dilutions of virus in Dulbecco’s Modified Eagle Medium D-glucose (1 g/L) and sodium pyruvate/L-Glutamine (100 ng/L) (Life Technologies Australia, Mulgrave, Australia). The virus was allowed to adsorb for 2 h at 37 °C, then cells were overlayed with 8% *w*/*v* carboxy-methyl cellulose (Sigma-Aldrich, Castle Hill, Australia) in Medium 199 (Sigma-Aldrich). Plates were incubated at 37 °C and 5% *v*/*v* CO_2_/air for 5–7 days. At the conclusion of the incubation period, the overlay was discarded, and the cell monolayers were washed in PBS, dried and fixed with ice-cold (1:1 (*v*/*v*) acetone-methanol (200 µL/well) at room temperature for 5 min. The fixative then was aspirated, and the plates dried at room temperature again for 1 h. Plates were stored inverted for at least 8 h at 4 °C before staining. Non-specific binding of antibodies to the cell monolayer was blocked by the addition of 200 µL of 5% *w*/*v* skim milk powder in PBS for 1 h at 37 °C. DENV infected cells were detected by the addition of 200 µL anti-flavivirus envelope reactive monoclonal antibody 4G2 (TropBio, Cairns, Australia) [48] diluted 1:1000 in 5% (*w*/*v*) skim milk powder in 1× Phosphate Buffered Saline (PBS) to each well for 1 h at 37 °C. After six washes with PBS, horseradish peroxidase (HRP)-conjugated goat anti-mouse IgG (Invitrogen, Carlsbad, CA, USA) diluted 1:2000 *v*/*v* in 5% *w/v* SMP/PBS was added to each cell monolayer for 1 h at 37 °C. The antibody solution then was discarded, the cell monolayers washed six times with PBS and 200 µL of substrate/chromogen (urea hydrogen peroxide/3,3′Diaminobenzidine, Sigma-Aldrich) added and incubated in the dark for 15 min. DENV positive foci or plaques were counted and stock concentration of each virus was quantified as plaque forming units (PFU) per mL.

### 5.4. Mosquito DENV Challenge Experiments

Mosquito eggs were hatched by submerging in aged de-chlorinated tap water (*Ae. aegypti*) or rainwater (*Ae. albopictus*). Larvae were reared at a density of 250 mosquitoes in 3 L of water in larval development trays and were provided fish food (Tetramin, Tetra Melle, Germany) *ad libitum*. Pupae were transferred to 500 mL bowls inside 30 × 30 × 30 cm insect rearing cages (BugDorm, Megaview, Taiwan) where adults emerged and mated freely. Adults were maintained on 10% *w/v* sugar water until blood feeding.

Female mosquitoes (5–7 days old) were starved for 12 h and deprived of water for 6 h prior to being offered a viraemic blood meal for 1 h on a membrane. For each mosquito species, two groups of approximately 100 mosquitoes each were exposed to one strain of DENV. The membrane feeding apparatus consisted of a series of glass membrane feeders with inner blood-filled chambers covered with pig intestinal membrane (sausage casing) and outer chambers connected by pipes circulating water from a 37 °C water bath. The blood meal consisted of 1:1 mix of defibrinated sheep blood (Equicell, Melbourne, Australia) and virus (~10^7^ PFU/mL). Adenosine Triphosphate (ATP) (Sigma-Aldrich) was added to the blood mixture to a final concentration of 5 Mm. Sub-samples of the blood meal were saved at the completion of feeding to determine the titre of infectious virus at the end of the feeding process. No significant decreases in the titres of virus were observed (data not shown). After 1 h of feeding, mosquitoes were anaesthetized with CO_2_ and sorted on a cold table within a Perspex glove box. Mosquitoes that were not completely engorged were discarded. The engorged mosquitoes for each mosquito virus combination were transferred to 250 mL plastic cups and held for up to 14 days in environmental chambers at 27 (±1) °C with 12 h-12 h light-dark (L:D) cycles and provided with 10% *w*/*v* sugar water *ad libitum.*

For each mosquito-virus combination, 20 mosquitoes were harvested at each of four time points (3, 6, 10 and 14 days) post blood feeding by anaesthetizing the insects with CO_2_ and then with ice before removing legs and wings. The bodies were stored separately from the legs/wings in microtubes and preserved at −80 °C until further testing.

### 5.5. Detection of Dengue Virus in Mosquito Tissue by qRT-PCR

Mosquito tissue samples were thawed on ice and total RNA isolated as previously described [49]. Briefly, each sample was homogenized with 100-µL extraction buffer (10 mM Tris pH 8.2, 1 mM EDTA, 50 mM NaCl) and 60 µL proteinase K (15 mg/mL Bioline) in a mini-beadbeater (Biospec, Bartlesville, OK, USA) for 1.5 min and then incubated in a heat block for 5 min at 56 °C. The sample then was heated at 98 °C for 5 min (to inactivate proteinase K). One-step qRT-PCR was carried out in 384-well Hard-Shell^®^ thin-wall plates (Bio-Rad, Gladesville, Australia) with the CFX384™ real-time PCR detection system (Bio-Rad). The reaction mix was prepared using Taqman^®^ Fast Virus One-Step RT-PCR Master Mix Reagents (Applied Biosystems, Thermo Fisher Scientific, Brisbane, Australia), following the manufacturer’s instructions. Each sample was assayed in a 10 µL reaction volume consisting of 2.5 µL of Taqman^®^ Fast Virus One-Step mix, 3 µL of total RNA extract and 400 nM each of the forward and reverse primers and 250 nM of probe labelled with FAM and BHQ-1 at the 5′ and 3′ ends respectively. Reverse transcription (RT) was performed at 50 °C for 3 min, followed by inactivation and denaturation of RNA-DNA hybrids at 95 °C for 20 s. This was followed by 45 cycles of 95 °C for 10 s, 60 °C for 30 s and 72 °C for 1 s. A plasmid containing the target DENV genome sequence (synthesized at Genscript, Piscataway, NJ, USA), serially diluted to known concentrations, was used to produce DNA standards. Ten-fold dilutions (10^7^ to 10^1^ DNA copies/µL) of the purified linearized plasmids were tested to generate standard curves and to determine the limit of detection across qPCR runs.

To determine the threshold cycle (Ct), the threshold level of fluorescence was optimized so that the standard curve gradient was the theoretical value of −3.30, which indicates 100% PCR efficiency. Data were accepted if the slope of the standard curve was between −3.0 and −3.6 and with a correlation coefficient (*r*^2^) above 0.95. The limit of detection was 10 genome copies/µL. All samples were analysed in triplicate with each analysis including positive and negative controls, non-template control (NTC) and a serially diluted standard curve of the plasmid standard (10^7^ to 10^1^ copies/µL). Samples with calculated DNA copy number values on or above the limit of detection were scored as “positive” indicating the presence of DENV.

### 5.6. Statistical Analysis

Infection was inferred to have occurred if virus was detected in the body. Dissemination was inferred to have occurred if the virus was detected in legs/wings. The infection rate was calculated as the number of infected mosquitoes divided by the number of mosquitoes engorged after the bloodmeal. The dissemination rate was calculated as the number of mosquitoes with legs/wings infected with DENV divided by the number of mosquitoes engorged after the blood meal. Infection rates were compared using Fisher’s Exact tests. Duplicate estimates of the DENV RNA loads per sample were summarised by the geometric mean and then were transformed to be on the log_10_ scale. For samples where all technical replicates were negative (i.e., below the limit of detection) were given a value of 1, so that when analysis on the log10 scale was performed they would have a value of 0. A general linear model (GLM) was performed on the data. Preliminary analysis indicated similar variances between mosquitoes within 64 combinations (8 virus strains × 2 mosquito species × 4 time points) per body site (body or legs/wings), such that the 20 mosquitoes per combination could be pooled together and summarised as the mean of the log_10_ RNA data. Preliminary GLMs indicated that the 2-way and 3-way interactions effects were of similar size and could be pooled as an estimate of error to test for differences between main effects. Hence the GLM tested for differences between levels of the main effects as a higher order of magnitude than the interactions. Therefore, the following GLM was applied to the mean of the log_10_ RNA data and performed separately for the body and legs/wings data.

Log_10_(RNA) = *β_Day_Day* + *β_Sp_Species* + *β_V_Virus*

The model had three main effects: Day being a categorical variable of the time point (3, 6, 10 or 14 d). Species being a categorical variable of the two mosquito species (*Ae. aegypti* and *Ae. albopictus*), and Virus being a categorical variable of the eight different dengue virus strains.

Statistical analyses of infectivity and dissemination rates were conducted with STATA version 15, analyses and graphs of DENV RNA loads were conducted in R (Version 3.6.2) Pairwise comparisons for Virus type and Day were performed using Tukey’s method in the glht function in R. Graphs prepared with GraphPad Prism^®^ Version 7.00 (GraphPad Software, La Jolla, CA, USA, 2008).

## Figures and Tables

**Table 1:** Strains of DENV used in vector competence experiments.

Serotype	Strain	Country of Origin	Date of Isolation
DENV-1	NC483	New Caledonia	2008
	ET243 *	Timor-Leste	2013
DENV-2	VN130604	Vietnam	2002
	55763	Timor-Leste	1985
DENV-3	ET-3 *	Timor-Leste	2000
DENV-4	31298	Cook Islands	1988
	MY1261	Myanmar	2000
	NC-39	New Caledonia	2009

* Strains were isolated in Australia from patients infected in Timor-Leste.

**Table 2:** Infection rates among *Aedes aegypti* and *Aedes albopictus* mosquitoes following feeding on eight strains of dengue from four serotypes. dpe, days post exposure. nt, not tested. *Ae. aegypti* mosquitoes infected with DENV-1 (ET-483) did not survive to 14 dpe.

			Mosquito Infection Rate (n Tested) at Various Days Post Exposure (dpe) to Dengue Virus							
			3 dpe		6 dpe		10 dpe		14 dpe	
Mosquito	Serotype	Strain	Bodies	Legs/Wings	Bodies	Legs/Wings	Bodies	Legs/Wings	Bodies	Legs/Wings
*Ae. aegypti*	DENV-1	NC-483	15 (20)	0 (20)	40 (20)	25 (20)	50 (20)	25 (20)	70 (20)	25 (20)
		ET-243	0 (20)	0 (20)	5 (20)	0 (20)	30 (20)	25 (20)	nt	nt
	DENV-2	VN-130604	5 (20)	0 (20)	10 (20)	5 (20)	45 (20)	40 (20)	35 (20)	20 (20)
		55763	5 (20)	0 (20)	30 (20)	10 (20)	30 (20)	20 (20)	40 (20)	25 (20)
	DENV-3	31298	5 (20)	0 (20)	5 (20)	5 (20)	10 (20)	5 (20)	25 (20)	20 (20)
		ET-3	0 (20)	0 (20)	0 (20)	0 (20)	20 (20)	5 (20)	25 (20)	10 (20)
	DENV-4	NC-39	0 (20)	0 (20)	5 (20)	0 (20)	10 (20)	5 (20)	30 (20)	10 (20)
		MY-1261	5 (20)	0 (20)	15 (20)	10 (20)	25 (20)	20 (20)	25 (20)	20 (20)
*Ae. albopictus*	DENV-1	NC-483	0 (20)	0 (20)	40 (20)	20 (20)	40 (20)	20 (20)	55 (20)	30 (20)
		ET-243	0 (20)	0 (20)	0 (20)	0 (20)	15 (20)	10 (20)	25 (20)	15 (20)
	DENV-2	VN-130604	5 (20)	0 (20)	10 (20)	0 (20)	35 (20)	30 (20)	40 (20)	30 (20)
		55763	5 (20)	0 (20)	15 (20)	10 (20)	25 (20)	10 (20)	20 (20)	10 (20)
	DENV-3	31298	0 (20)	0 (20)	0 (20)	0 (20)	10 (20)	10 (20)	45 (20)	20 (20)
		ET-3	0 (20)	0 (20)	0 (20)	0 (20)	10 (20)	5 (20)	40 (20)	10 (20)
	DENV-4	NC-39	0 (20)	0 (20)	0 (20)	0 (20)	0 (20)	0 (20)	10 (20)	5 (20)
		MY-1261	10 (20)	0 (20)	10 (20)	10 (20)	15 (20)	10 (20)	15 (20)	10 (20)

**Table 3:** Main effects of virus strain, incubation period and mosquito species on the number of DENV genome copies in mosquito bodies and legs/wings determined using a general linear model (GLM). Data excludes samples in which no DENV copies were detected. The main effects that are significant at *p*-value < 0.05 are bolded. df, degrees of freedom. F, F statistic.

Effect	Body (Error df = 37)			Legs/Wings (Error df = 26)		
	df	F	*p*-Value ^†^	df	F	*p*-Value ^†^
Incubation period	**3**	**4.09**	**0.013**	2 *	**19.66**	**<0.001**
Mosquito Species	1	0.48	0.49	1	2.96	0.097
Virus strain	**7**	**4.12**	**0.002**	**7**	**4.77**	**0.001**

* Legs/wings sampled at 3 dpe were negative for DENV. ^†^ *p*-value based on the F-statistic with degrees of freedom corresponding to the effect and the error term.

## Supplementary Materials

The following are available online at https://www.mdpi.com/2076-0817/9/8/668/s1, Table S1: Variation in infection and dissemination rates between dengue serotypes in Ae. aegypti. Percentage (number tested) of mosquitoes with detectable dengue infection in bodies or legs and wings at various times after feeding virus from one of the four DENV serotypes. Comparisons were made using Fisher’s Exact test. † Mosquitoes infected with DENV-1 strain NC-483 did not survive to 14 d and therefore DENV-1 was excluded from the analysis of samples at this time point. Table S2: Variation in infection and dissemination rates between dengue serotypes in Ae. albopictus. Percentage (number positive/number tested) of Ae. albopictus mosquitoes with detectable dengue infection in bodies or legs and wings at various times after feeding on virus from one of the four DENV serotypes. Comparisons were made using Fisher’s Exact test.
