# Supplementary material for: Effect of Serotype and Strain Diversity on Dengue Virus Replication in Australian Mosquito Vectors
Source: Pathogens. 2020 Aug 18;9(8):668. doi: 10.3390/pathogens9080668 (PMC7460537; doi:10.3390/pathogens9080668)
Supplement: Supplementary file 1 [file pathogens-09-00668-s001.pdf]

Supplementary Table S1. Variation in infection and dissemination rates between dengue serotypes in *Ae. aegypti*. Percentage (number tested) of mosquitoes with detectable dengue infection in bodies or legs and wings at various times after feeding virus from one of the four DENV serotypes. Comparisons were made using Fisher's Exact test. † Mosquitoes infected with DENV-1 strain NC-483 did not survive to 14 d and therefore DENV-1 was excluded from the analysis of samples at this time point.

**Serotype**	**3 dpe**		**6 dpe**		**10 dpe**		**14 dpe**	
	**Bodies**	**Legs/Wings**	**Bodies**	**Legs/Wings**	**Bodies**	**Legs/Wings**	**Bodies**	**Legs/Wings**
DENV-1	7.5 (40)	0 (40)	22.5 (40)	12.5 (40)	40.0 (40)	27.5 (40)	nt †	Nt †
DENV-2	5.0 (40)	0 (40)	20.0 (40)	7.5 (40)	50.0 (40)	27.5 (40)	37.5 (40)	22.5 (40)
DENV-3	2.5 (40)	0 (40)	2.5 (40)	2.5 (40)	15.0 (40)	5.0 (40)	25.0 (40)	15.0 (40)
DENV-4	2.5 (40)	0 (40)	10.0 (40)	5.0 (40)	17.5 (40)	10.0 (40)	27.5 (40)	15.0 (40)
p-value	**0.84**	**NA**	**0.024**	**0.41**	**0.016**	**0.008**	**0.538†**	**0.72†**

Supplementary Table S2. Variation in infection and dissemination rates between dengue serotypes in *Ae. albopictus*. Percentage (number positive / number tested) of *Ae. albopictus* mosquitoes with detectable dengue infection in bodies or legs and wings at various times after feeding on virus from one of the four DENV serotypes. Comparisons were made using Fisher's Exact test.

**Serotype**	**3 dpe**		**6 dpe**		**10 dpe**		**14 dpe**	
	**Bodies**	**Legs/Wings**	**Bodies**	**Legs/Wings**	**Bodies**	**Legs/Wings**	**Bodies**	**Legs/Wings**
DENV-1	0 (0/40)	0 (0/40)	20.0 (8/40)	10.0 (4/40)	27.5 (11/40)	15 (6/40)	40.0 (16/40)	22.5 (9/40)
DENV-2	5.0 (2/40)	0 (0/40)	12.5 (5/40)	2.5 (1/40)	30.0 (12/40)	20 (8/40)	30.0 (12/40)	20.0 (8/40)
DENV-3	0 (0/40)	0 (0/40)	0 (0/40)	0 (0/40)	10.0 (4/40)	5 (2/40)	42.5 (17/40)	27.5 (11/40)
DENV-4	5.0 (2/40)	0 (0/40)	5.0 (2/40)	5.0 (2/40)	7.5 (3/40)	2.5 (1/40)	12.5 (5/40)	7.5 (3/40)
p-value	**0.33**	**NA**	**<0.001**	**0.012**	**0.014**	**0.019**	**0.011**	**0.11**
